# Non-coding RNA and drug resistance in head and neck cancer

**DOI:** 10.20517/cdr.2024.59

**Published:** 2024-09-20

**Authors:** Yulong Zhang, Yingming Peng, Bingqin Lin, Shuai Yang, Feiqiang Deng, Xuan Yang, An Li, Wanyi Xia, Chenxi Gao, Shaona Lei, Wei Liao, Qi Zeng

**Affiliations:** ^1^Cancer Center, The Fifth Affiliated Hospital of Sun Yat-sen University, Zhuhai 519000, Guangdong, China.; ^2^Key Laboratory of Traditional Chinese Medicine for the Prevention and Treatment of Infectious Diseases, Traditional Chinese Medicine Bureau of Guangdong Province, the Fifth Affiliated Hospital, Sun Yat-sen University, Zhuhai 519000, Guangdong, China.; ^3^Department of Radiotherapy and Minimally Invasive Surgery, Cancer Center, The Fifth Affiliated Hospital of Sun Yat-sen University, Zhuhai 519000, Guangdong, China.; ^4^Department of Otolaryngology and Head and Neck Surgery, The Fifth Affiliated Hospital of Sun Yat-sen University, Zhuhai 519000, Guangdong, China.; ^#^Authors contributed equally.

**Keywords:** Head and neck cancer, non-coding RNA, microRNA, long non-coding RNA, circular RNA, drug resistance

## Abstract

Head and neck cancer (HNC) is ranked as the sixth most common malignant tumor, and the overall survival rate with current treatment options remains concerning, primarily due to drug resistance that develops following antitumor therapy. Recent studies indicate that non-coding RNAs play a crucial role in drug resistance among HNC patients. This article systematically reviews the current research landscape, explores novel targets and treatment strategies related to non-coding RNAs and HNC resistance, raises some unresolved issues, and discusses five promising research directions in this field: ferroptosis, nanomedicine, exosomes, proteolysis-targeting chimeras (PROTACs), and artificial intelligence. We hope that our work will contribute to advancing research on overcoming HNC resistance through the regulation of non-coding RNAs.

## INTRODUCTION

Head and neck cancer (HNC) ranks as the sixth most prevalent malignant neoplasm, presenting significant challenges in terms of treatment^[1]^. It affects various anatomical sites including the lips, oral cavity, pharynx, larynx, nose, salivary glands, and thyroid. Squamous cell carcinoma (SCC) and its variants constitute more than 90% of the histopathological types^[2]^. Major risk factors associated with HNC include tobacco and alcohol use, as well as infections with human papillomavirus (HPV) and Epstein-Barr virus (EBV)^[3]^. Globally, the incidence of HNC has been gradually declining, primarily attributed to the reduction in tobacco use and lifestyle modifications^[4,5]^.

Treatment decisions are guided by the precise location, stage, and pathologic characteristics of the disease. For around 30%-40% of patients diagnosed with early-stage disease (Stage I or II), the typical recommendation involves a single treatment modality such as surgery or radiotherapy. Conversely, roughly 60% of patients presenting with locally or regionally advanced disease upon diagnosis typically receive a multidisciplinary approach encompassing a combination of treatments. These may involve surgery, radiotherapy, chemotherapy, and immunotherapy, as well as additional measures such as nutritional support, psychological counseling, supportive care, and rehabilitation^[2]^. Various studies have indicated that the overall 5-year survival rate for individuals with HNC falls below 50%, underscoring the persisting challenges in current treatment outcomes^[2,6]^. The inadequate response of patients to antitumor therapy, and in some cases, the development of drug resistance, contribute to unsatisfactory clinical outcomes and stand as significant factors leading to mortality^[7]^.

Unfortunately, the key determinants underlying this resistance phenomenon remain largely elusive. Advances in molecular biology and gene sequencing have unveiled that approximately 98% of human DNA is designated non-protein coding^[8]^. Non-coding RNAs constitute a diverse group of RNA transcripts that lack protein-coding potential. Significant subtypes include microRNAs (miRNAs), circular RNAs (circRNAs), and long non-coding RNAs (lncRNAs)^[9]^. Emerging research has revealed the potential of non-coding RNAs to modulate multiple facets of cellular functions, encompassing growth, proliferation, differentiation, development, metabolism, infection, immunity, cell death, organelle biogenesis, messenger signaling, DNA repair, and self-renewal^[10-12]^. Moreover, non-coding RNAs exhibit close associations with various common diseases, particularly cancer^[9,13-15]^. Additionally, non-coding RNAs function as widespread regulators of various cancer-related characteristics, including proliferation, apoptosis, invasion, metastasis, and genomic instability, thus playing a crucial role in mediating resistance to different cancer therapies^[16,17]^. Consequently, gaining a comprehensive understanding of the mechanisms underlying non-coding RNAs and drug resistance holds significant importance in the context of HNC treatment. In addition, the research on overcoming tumor drug resistance through the regulation of non-coding RNAs offers many advantages^[16,18-28]^ [Table 1].

**Table 1:** Advantages of targeting non-coding RNAs to overcome tumor drug resistance

**Aspect**	**Summary**	**Ref.**
Complexity of regulatory networks	ncRNAs form intricate regulatory networks that influence various biological processes, including gene expression, signal transduction, and the cell cycle. Targeting these nodes can impact tumor growth and drug resistance	[18]
Specific role	ncRNAs may be uniquely expressed in tumor cells or play critical roles in tumor development and drug resistance, enabling the development of targeted therapies	[19]
Multi-target intervention	ncRNAs can affect multiple signaling pathways and biological processes simultaneously, making them suitable for multi-target interventions to address multidrug resistance	[20]
Drug tolerance	ncRNAs may have a lower risk of tolerance development compared to protein targets due to their distinct regulatory mechanisms	[21]
Diverse treatment strategies	ncRNAs can act as direct drug targets, carriers, or therapeutic agents, offering a range of treatment strategies	[16]
New drug discovery	Research on ncRNAs provides new molecular targets for drug development, aiding in the discovery of novel antitumor agents	[22]
Personalized medicine	ncRNA-based biomarkers can guide personalized treatment plans, helping physicians select the most suitable therapy for individual patients	[23-25]
Combination therapy	ncRNAs can be combined with other therapies (e.g., chemotherapy, radiotherapy, immunotherapy) to improve efficacy and reduce drug resistance	[26]
Treatment monitoring and evaluation	ncRNA expression levels can be used as biomarkers to monitor treatment efficacy, tumor response, and drug resistance development	[27]
Gene editing applications	Gene editing technologies like CRISPR/Cas9 offer tools for targeting ncRNAs, enabling precise regulation of their expression and influencing drug resistance	[28]

ncRNAs: Non-coding RNAs.

This review aims to provide a comprehensive summary of the intricate relationship between non-coding RNAs and drug resistance in HNC. It delves into the various types of non-coding RNAs and their potential roles in mediating resistance to different therapeutic approaches utilized in HNC treatment. Through an analysis of the current literature, this review seeks to enhance our understanding of the mechanisms underlying drug resistance in HNC and identify potential avenues for improving treatment outcomes.

## miRNAs IN HNC CELL DRUG RESISTANCE

miRNAs, approximately 22 nucleotides in length, constitute a class of endogenous non-coding RNAs. These molecules regulate diverse biological processes, including cell proliferation, differentiation, and apoptosis, through specific binding to the 3’-untranslated region (3’-UTR) of target gene mRNAs, thereby inducing mRNA degradation or inhibiting translation^[29-31]^. Numerous studies have demonstrated the involvement of miRNA dysregulation in the development and chemoresistance of HNC^[32-34]^.

### miRNAs dysregulation is closely related to chemoresistance in HNC

Research indicates a close association between miRNA dysregulation and chemoresistance in HNC. Zhang *et al*. observed the downregulation of miR-216a-5p and ZEB1 in laryngeal squamous cell carcinoma (LSCC) tissues. Moreover, they demonstrated that overexpression of miR-216a-5p could reverse the malignant phenotype and cisplatin (CDDP) resistance of LSCC cells by targeting ZEB1^[35]^. Gao *et al*. identified that circ_0109291 promoted CDDP resistance in oral squamous cell carcinoma (OSCC) cells. They elucidated its mechanism by showing that circ_0109291 sponged miR-188-3p, leading to upregulation of ABCB1 expression^[36]^. Cao *et al*. revealed that HOTAIR induced CDDP resistance in nasopharyngeal carcinoma (NPC) by sponging miR-106a-5p, consequently upregulating SOX4 expression^[37]^. The miR-200 family constitutes a pivotal group of miRNAs implicated in the regulation of epithelial-mesenchymal transition (EMT). Their downregulation is closely linked to chemoresistance in HNC. For instance, reduced expression of Let-7, miR-200, and miR-203 correlates with docetaxel resistance in OSCC^[38-42]^. Moreover, the miR-200c/c-myc negative feedback loop orchestrates EMT, stemness, and chemoresistance in NPC^[43,44]^. Additionally, dysregulation of miR-138, miR-222, miR-101, miR-23a, miR-214, and various other miRNAs has been associated with chemoresistance in HNC^[45-47]^. In summary, the dysregulation of specific miRNAs plays a key role in the acquired chemoresistance of HNC.

### miRNAs affect chemoresistance by regulating autophagy signaling pathways

Autophagy and miRNAs are intricately linked to chemoresistance in HNC. H19 induces CDDP resistance in LSCC cells by upregulating the expression of autophagy-related proteins Atg5 and Beclin1 through the miR-107/HMGB1 axis^[48]^. MiR-155 inhibitor-loaded exosomes reverse CDDP resistance in OSCC by inducing autophagy through the upregulation of FOXO3a^[49]^. Yang *et al*. found that upregulated miR-214 expression could inhibit autophagy in OSCC cells by targeting autophagy-related genes ULK1 and ATG5, consequently enhancing chemosensitivity^[50]^. CircAP1M2 promotes CDDP resistance and autophagy in OSCC by inhibiting miR-1249-3p to upregulate ATG9A expression^[51]^. MiR-1278 inhibits autophagy and CDDP resistance in NPC cells by targeting ATG2B^[52]^. Therefore, targeting autophagy pathways through the regulation of miRNAs may enhance the therapeutic sensitivity of HNC.

### miRNAs affect chemoresistance by regulating the cell cycle and metabolism

miRNAs can also modulate the sensitivity of tumor cells to treatment by regulating energy metabolism and cell cycle. Fan *et al*. discovered that downregulation of mitochondrial miR-2392 expression could lead to impaired mitochondrial oxidative phosphorylation by inhibiting MT-CO3 translation, resulting in a shift of glucose metabolism from oxidative phosphorylation to anaerobic glycolysis, ultimately causing CDDP resistance in tongue squamous cell carcinoma (TSCC)^[53]^. Chen *et al*. discovered that miR-5787 contributes to CDDP resistance in TSCC by reprogramming glucose metabolism via inhibiting the translation of MT-CO3^[54]^. In another study, Mortezagholi *et al*. demonstrated that miR-34 could reverse paclitaxel resistance in OECM-1 oral cancer cells by inducing DNA damage and apoptosis in a p53-dependent manner^[55]^. HOXA11-AS enhances the proliferation, invasion, survival, and drug resistance of OSCC by sponging miR-494 to promote NQO1 expression and recruiting EZH2 to the NQO2 promoter to suppress NQO2 expression^[56]^. Kang *et al*. demonstrated that cancer-associated fibroblast (CAF)-derived extracellular vesicles carrying miR-876-3p can modulate CDDP resistance in OSCC by targeting insulin-like growth factor binding protein 3 (IGFBP3)^[57]^. Li *et al*. identified miR-194 as a tumor suppressor in LSCC that inhibits cell proliferation and enhances chemosensitivity by targeting Wee1^[58]^.

### miRNAs affect chemoresistance via exosome-mediated mechanisms

Exosomes, as an important mediator of intercellular communication, can carry functional molecules such as miRNAs to mediate information exchange between tumor cells and the microenvironment, playing a key role in tumor progression and chemoresistance^[59-61]^. Qin *et al*. found that CAF-derived exosomal miR-196a could confer CDDP resistance to head and neck squamous carcinoma cells by targeting CDKN1B and ING5^[62]^. Li *et al*. discovered that exosomal miR-106a-5p derived from CDDP-resistant cells could promote NPC cell proliferation and suppress apoptosis by targeting ARNT2 and activating AKT phosphorylation, thereby regulating tumorigenesis^[63]^. This study revealed that tumor cells could remodel the tumor microenvironment through exosomal miRNAs to acquire a chemoresistant phenotype.

### miRNAs affect chemoresistance by regulating cancer stem cells

Cancer stem cells play a key role in tumor development, metastasis, and chemoresistance^[64-66]^. MiRNAs are involved in the chemoresistance process of HNC by regulating cancer stemness. Recent studies have shown that miRNA-485-5p can regulate the stemness and chemotherapy resistance of OSCC by targeting keratin 17 (KRT17)^[67]^. MiR-21-3p overexpression maintained the stemness of this subpopulation. Lin *et al*. discovered that activation of the miR-371/372/373 cluster could enhance the tumorigenicity and drug tolerance of OSCC cells^[68]^. Cai *et al*. found that EBV-encoded miR-BART7-3p enhanced the stemness and chemotherapy resistance of NPC by inhibiting SMAD7 to activate the TGF-β pathway^[69]^.

### miRNAs affect chemoresistance by regulating other signaling pathways

In addition, miRNAs are also involved in chemoresistance of HNC by regulating multiple signaling pathways. Zhang *et al*. found that miR-205-5p could induce EMT and CDDP resistance in NPC by targeting PTEN to activate the PI3K/Akt pathway^[70]^. Gu *et al*. discovered that miR-552 could promote the proliferation and metastasis of laryngeal cancer cells by targeting p53^[71]^. Sheng *et al*. discovered that miR-21 enhances proliferation, apoptosis inhibition, and CDDP resistance in head and neck squamous cell carcinoma (HNSCC) through the PTEN/PI3K/AKT pathway^[72]^. Wu *et al*. found that the miR-577/EIF5A2 axis suppresses the proliferation of CDDP-resistant NPC by blocking the TGF-β signaling pathway and inhibiting EMT^[73]^. In a study by Chen *et al*., miR-132 was shown to inhibit proliferation and invasion while enhancing CDDP chemosensitivity in OSCC cells via the TGF-β1/Smad2/3 signaling pathway^[74]^. Furthermore, miRNA-296-5p was found to increase the sensitivity of NPC cells to CDDP by targeting and suppressing the STAT3/KLF4 signaling axis^[75]^. These results highlight specific miRNAs play a key role in the acquired chemoresistance of HNC by fine-tuning multiple signaling pathways.

### miRNAs regulate chemoresistance in HNC by competitively binding to upstream lncRNAs

miRNAs also regulate chemoresistance in HNC by competitively binding to upstream lncRNAs. FOXD1 promotes OSCC chemoresistance by upregulating LPP expression via sponging miR-1252-5p and miR-3148 through CYTOR^[76]^. PVT1 promotes cetuximab resistance in head and neck squamous carcinoma by inhibiting miR-124-3p^[77]^. KCNQ1OT1 promotes CDDP resistance in NPC through the miR-454/USP47 axis^[78]^. NEAT1 promotes CDDP resistance in thyroid cancer(TC) through the miR-9-5p/SPAG9 axis^[79]^. CircRNAs also regulate chemoresistance in HNC by sponging miRNAs, such as circCRIM1 promoting docetaxel resistance in NPC through the miR-422a/FOXQ1 axis^[80]^.

Furthermore, miRNAs can also be involved in the chemoresistance of HNC by affecting drug resistance-related enzymes, membrane transporters, angiogenesis, and other factors. Yuan *et al*. found that LINC-PINT could reverse laryngeal cancer stemness and chemotherapy resistance through the miR-425-5p/PTCH1/SHH axis^[81]^. MiR-340 reverses multidrug resistance in NPC by inhibiting P-gp and BCRP^[82]^. Docetaxel can induce IL-8 secretion in OSCC cells, thereby promoting angiogenesis.

**Figure 1:**
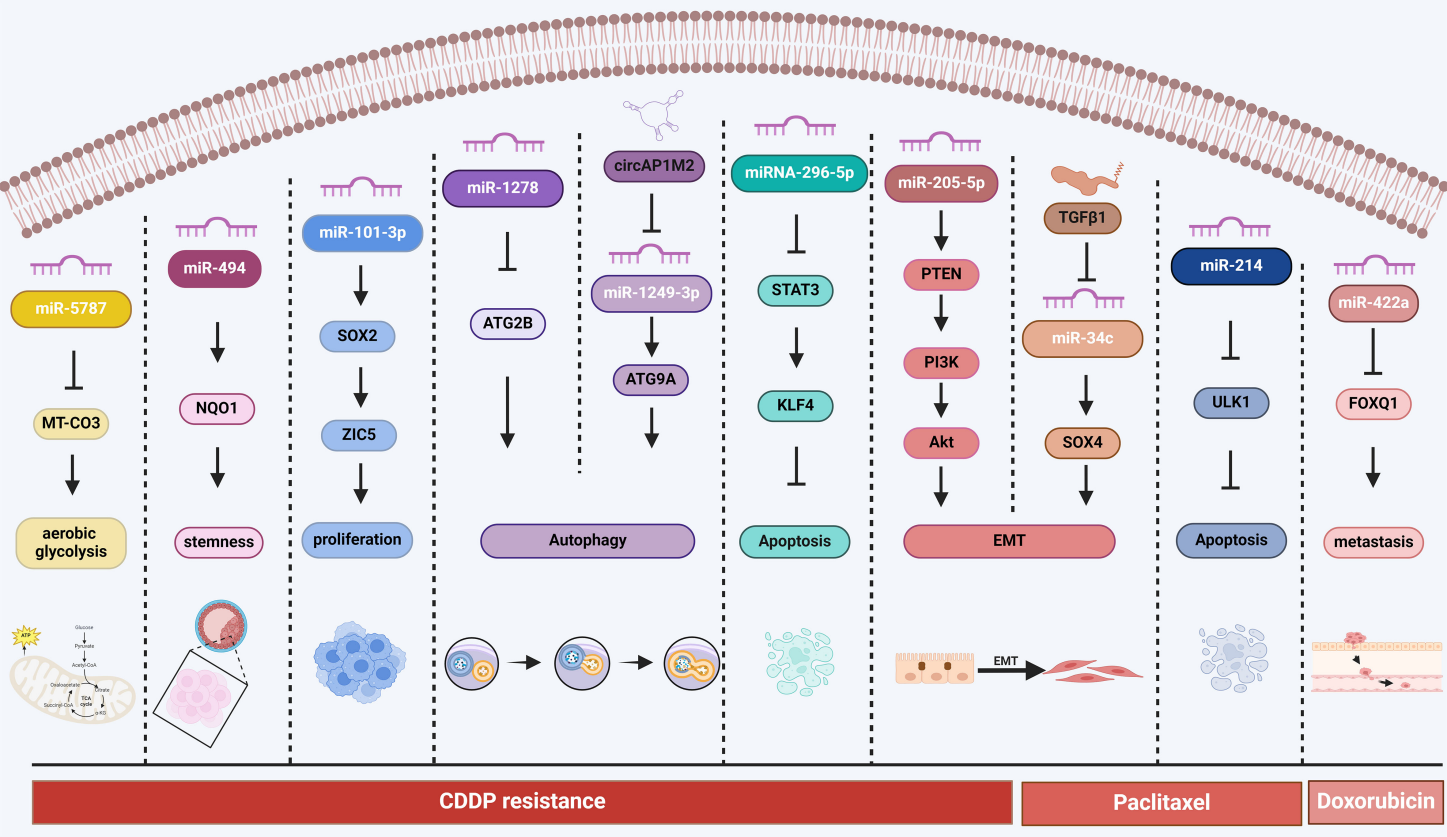
Overview of the main molecular mechanisms of microRNAs in HNC drug resistance. HNC: Head and neck cancer.

In recent years, strategies targeting miRNAs to reverse chemoresistance have received widespread attention. Li *et al*. found that miR-101-3p mimics could inhibit the proliferation and CDDP resistance of NPC cells through ZIC5^[47]^. Song *et al*. discovered that miR-619-5p inhibitors could enhance the CDDP sensitivity of OSCC cells by activating ATXN3^[83]^ [Table 2 and Figure 1]. Although miRNAs have shown good antitumor and chemoresistance reversal effects at the animal level, their clinical application still faces many challenges, such as the construction of miRNA delivery systems, in vivo stability, and off-target effects^[84-87]^. In addition, the role of circRNAs and lncRNAs as endogenous miRNA sponges in reversing chemoresistance requires further in-depth research.

**Table 2:** HNC cell drug resistance-related microRNAs

**Tumor type**	**MiRNAs**	**Cell line**	**Expression level**	**Target**	**Functions**	**Corresponding drugs**	**Ref.**
NPC	miR-106a-5p	C666-1, CNE2	Down	SOX4	Enhance DDP resistance in NPC cells	CDDP	[37]
NPC	miR-200c	CNE1, CNE2, 5-8F	Up	c-Myc	Promising approach to overcome the oncogenic role of c-Myc in NPC	Cisplatin	[43]
NPC	miR-101-3p	HNE-1/DDP, C666-1/DDP	Up	SOX2	Inhibit cisplatin resistance through miR-101-3p/SOX2/ZIC5 axis	CDDP	[47]
NPC	miR-1278	CNE-1, CNE-2, C666-1, 5-8F, HONE-1	Up	ATG2B	Sensitize NPC cells to DDP and reduce autophagy	CDDP	[52]
NPC	miR-106a-5p	CNE1	Down	ARNT2	Improve recipient cell proliferation, metastasis, and chemoresistance	CDDP	[63]
NPC	miR-205-5p	HNE1/DDP	Down	PTEN	Restrain EMT progression of HNE1/DDP cells	CDDP	[70]
NPC	miR-454	5-8F, SUNE-1	Down	USP47	Suppress NPC cell viability and DDP resistance	CDDP	[78]
NPC	miR-422a	S18, S26	Down	FOXQ1	Promote NPC cell metastasis and EMT	CDDP	[80]
NPC	miR-181a	C666-1, SUNE1	Up	KDM5C	Delay NPC cell progression	/	[82]
LSCC	miR-216a-5p	Tu-686, SNU899, SNU46, Tu-177	Up	ZEB1	Downregulate the cell proliferation and migration and invasive ability	/	[35]
LSCC	miR-107	TU-177, AMC-HN-8	Down	HMGB1	Increase cisplatin resistance	CDDP	[48]
LSCC	miR-425-5p	Hep-2	Up	PTCH1	Regulate laryngeal carcinoma cells through miR‐425‐5p/PTCH1	CDDP	[81]
OSCC	miR-188-3p	SCC-4, SCC-9, CAL-27, UM1, UM2	Down	ABCB1	Promote cisplatin resistance of Oral Squamous Cell Carcinoma	CDDP	[36]
OSCC	miR-155	UPCI-SCC-131, UPCI-SCC-131R	Down	FOXO3a	Suppress the stem-cell-like property and drug efflux transporter protein expression	CDDP	[49]
OSCC	miR-1249-3p	CAL27, SCC15	Down	ATG9A	Promote autophagy and induce cisplatin resistance	CDDP	[51]
OSCC	miR-34	OECM-1/PTX	Up	p53	Increase DNA damage and apoptosis in a p53-depended manner	Paclitaxel	[55]
OSCC	miR-494	HSC3, HSC4	Down	NQO1	Induce drug resistance and increase stemness	CDDP	[56]
OSCC	miR-371/372/373	SAS subclones	Up	AKT, β-catenin and Src	Increase both oncogenicity and drug resistance	CDDP	[68]
OSCC	miR-1252-5p	CAL-27 and SCC4	Down	FOXD1	Upregulate LPP expression	CDDP	[76]
OSCC	miR-3148	CAL-27 and SCC4	Down	FOXD1	Upregulate LPP expression	CDDP	[76]
OSCC	miR-619-5p	HN6 and CAL27	Up	ATXN3	Inhibit proliferation and arrest cell cycle progression	CDDP	[84]
TC	miR-9-5p	Nthy-ori 3-1, SW1736, 8505C	Up	SPAG9	Sensitize ATC cells to DDP	CDDP	[79]
TSCC	miR-200c	HSC-3	Down	TUBB3 and PPP2R1B	Increase resistance to DTX, migration, and invasion and decrease apoptosis	Paclitaxel	[38]
TSCC	miR-214	CAL-27 and CP-H203	Up	ULK1	Antagonize antitumor effect	CDDP, paclitaxel	[50]
TSCC	miR-2392	CAL-27 and SCC-9	Up	AGO2	Inhibit apoptosis and cisplatin sensitivity	CDDP	[53]
TSCC	miR-5787	Cal27	Down	MT-CO3	Inhibit the translation of MT-CO3 to regulate cisplatin resistance	CDDP	[54]

HNC: Head and neck cancer; NPC: nasopharyngeal carcinoma; DDP: cisplatin; CDDP: cisplatin; LSCC: laryngeal squamous cell carcinoma; OSCC: oral squamous cell carcinoma; TC: thyroid cancer; TSCC: tongue squamous cell carcinoma; DTX: docetaxel.

## LONG NON-CODING RNAs IN HNC CELL DRUG RESISTANCE

LncRNAs are extensively expressed and have specific interactions with DNA, RNA, and proteins, allowing them to regulate chromatin function, modulate the assembly and function of membraneless nucleosomes, influence the stability and translation of cytoplasmic mRNA, and participate in the regulation of signaling pathways^[88,89]^. Moreover, lncRNAs play a crucial role in regulating processes such as apoptosis, drug efflux, drug metabolism, DNA repair, EMT, autophagy, and ferroptosis. Consequently, they emerge as pivotal mediators of tumor drug resistance^[90-93]^. In recent years, there has been a notable increase in the number of lncRNAs associated with cancer development and progression. These lncRNAs can be explored and investigated in meticulously curated databases like Lnc2Cancer3.0^[94]^ or Cancer LncRNA Census^[95]^.

### CDDP resistance

CDDP stands as a highly extensively employed drug in the treatment of diverse solid tumors^[96]^. Its principal mode of action involves disrupting DNA repair mechanisms, leading to DNA damage and consequently triggering apoptosis in cancer cells^[97,98]^. Nonetheless, drug resistance poses an inherent challenge in the clinical utilization of CDDP, primarily stemming from three molecular mechanisms: heightened DNA repair, modified cellular accumulation, and augmented drug inactivation^[99]^.In 2014, Galluzzi *et al*. Systematically categorized CDDP resistance into four distinct stages: Pre-Target, On-Target, Post-Target, and Off-Target^[100]^. In recent years, the advancement of molecular biology and multi-omics approaches has unveiled additional molecules and mechanisms associated with CDDP resistance in non-NHC. These include epigenetic mechanisms, as well as regulators such as HNRNPU, CHD4, TRPV1, MAFG-AS, and MAST1, among others^[101-106]^. Furthermore, a study demonstrated that in non-small cell lung cancer, MEK inhibitors successfully surmounted resistance to combination immunotherapy with CDDP by inducing CXCL10 expression in cancer cells^[107]^.

### lncRNAs mediate drug resistance of NPC

NPC represents an EBV-associated malignancy that is particularly common in southern China, Southeast Asia, and North Africa. The age-standardized incidence rate in these regions ranges from 4 to 25 cases per 100,000 individuals, as reported by GLOBOCAN^[5,108]^. Patients diagnosed with NPC generally exhibit a favorable prognosis, with a minimum 5-year survival rate of 80% in cases of locally advanced NPC. However, the 5-year survival rate drops to approximately 20% in recurrent or distant metastatic NPC^[109,110]^. The study found that CDDP-resistant NPC exhibits a high expression of lncRNA TINCR, which contributes to the development of chemoresistance via the TINCR/ACLY/PADI1/MAPK/MMP2/9 axis^[111]^. Another study identified elevated expression of LncRNA NEAT1 in NPC cells resistant to histone deacetylase inhibitors (HDACis). This upregulation is mediated by the NEAT1/miR-129/Bcl-2 axis, which plays a role in NPC's resistance to HDACis^[112]^. Consequently, an epigenetic therapeutic approach involving the use of HDACis in combination with other targeted agents holds promise as a novel strategy for future NPC treatment^[113,114]^.

### lncRNAs mediate drug resistance of HNC

The majority of HNCs originate from the mucosal epithelium of the oral cavity, pharynx, and larynx, making them the most prevalent malignant tumors in the head and neck region^[115]^. In head and neck squamous cell carcinoma (HNSCC), STAT3 promotes the transcription of lncRNA HOTAIR and its interaction with pEZH2-S21, which contributes to resistance against both CDDP and cetuximab^[116]^. Another study demonstrated that VN1R5 upregulates lncRNA POP1-1, leading to the promotion of CDDP resistance in HNSCC through its interaction with MCM5^[117]^. LSCC represents a highly prevalent subtype of laryngeal cancer, ranking second in incidence among respiratory tumors. Chemotherapy is regarded as the established first-line treatment for patients with advanced LSCC^[118]^. A study reported that the activation of the STAT3 signaling pathway by lncRNA FOXD2-AS1 enhances CDDP resistance in LSCC^[119]^. OSCC is the prevailing malignant tumor of the oral mucosa, with 57.5% of global oral cancer cases being reported in Asia, particularly in India^[120]^. FOXD1 upregulates the expression of lncRNA CYTOR, promoting EMT and conferring CDDP resistance in OSCC^[76]^. Additionally, it has been reported that in OSCC, HOXA11-AS upregulates the expression of NQO1 by sponging microRNA-494 while downregulating the expression of NQO2. This regulatory mechanism promotes tumor progression and contributes to drug resistance^[56]^. TSCC is characterized by a significant upregulation in the expression of miRNA processing-related lncRNA (MPRL), which disrupts pre-miRNA processing. This disruption promotes mitochondrial fission and enhances CDDP chemosensitivity in TSCC^[121]^. TC is a prevalent endocrine malignancy, comprising approximately 1% of all malignant tumors. Moreover, its incidence is consistently rising on a global scale^[122]^. Papillary thyroid cancer (PTC) is the most common histological subtype of TC, accounting for 89.1% of cases. Studies have shown that the LncRNA Glycolysis-Associated Regulator of LDHA post-transcriptional modification (GLTC) is significantly upregulated in PTC tissues and correlates with more extensive distant metastasis, increased tumor size, and poorer prognosis. GLTC facilitates the succinylation-dependent activation of LDHA, thus promoting resistance to radioiodine therapy. These findings provide a theoretical foundation for considering the GLTC-LDHA pathway a potential target for therapeutic intervention in PTC^[123]^.

**Figure 2:**
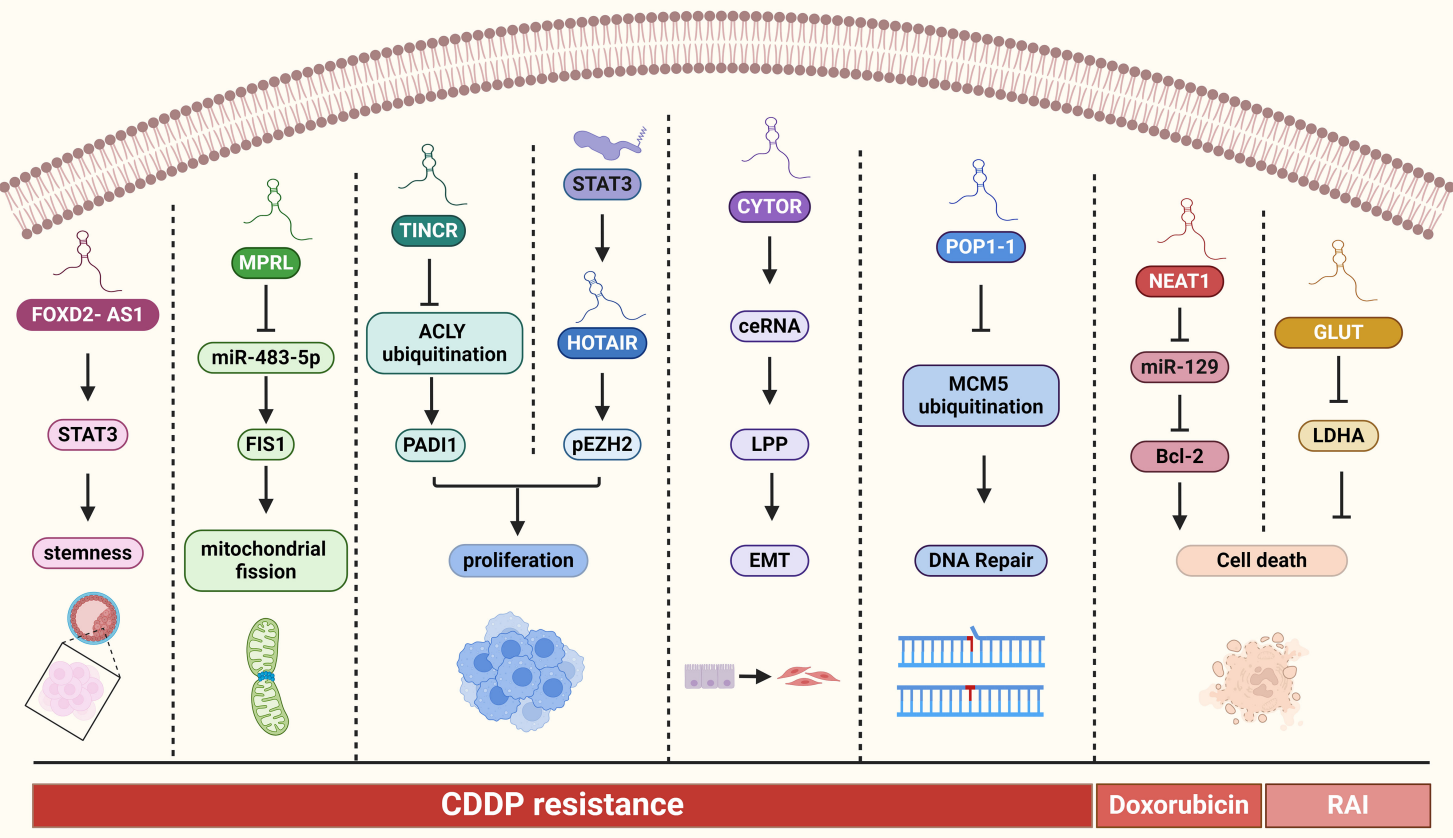
Overview of the main molecular mechanisms of lncRNAs in HNC drug resistance. HNC: Head and neck cancer.

Several lncRNAs have been found to have close associations with the malignant biology of tumors. In LSCC, HCP5 exhibits high expression levels. The knockdown of HCP5 has been shown to inhibit malignant biological functions through the regulation of miR-216a-5p/ZEB1 signaling pathway^[35]^. In OSCC, lnc-p23154 is believed to play a significant role in glut1-mediated glycolysis and promote tumor metastasis by suppressing the transcription of miR-378a-3p^[124]^. Additionally, the mitochondria-localized lncRNA growth-arrest-specific 5 (GAS5) has been identified as a tumor suppressor that plays a critical role in maintaining cellular energy homeostasis^[125]^. The targeting of epidermal growth factor receptor (EGFR) has proven to be an effective therapeutic strategy for the treatment of SCCs. Notably, studies have indicated that lnc-EGFR-AS1 is involved in mediating EGFR addiction and influencing treatment responses in SCCs^[126]^ [Table 3 and Figure 2].

**Table 3:** HNC cell drug resistance-related lncRNAs

**Tumor type**	**lncRNA**	**Cell line**	**Expression level**	**Target**	**Functions**	**Corresponding drugs**	**Ref.**
NPC	TINCR	NP69 N2Tert CNE-1, HK-1	Up	ACLY	Activate TINCR-ACLY-PADI1-MAPK-MMP2/9 axis	CDDP	[111]
NPC	NEAT1	C666-1, CNE-1, CNE-2	Up	miR-129	Modulate the miR-129/Bcl-2 axis	HDACis	[112]
HNSCC	HOTAI	SCC25, Cal27, UM1	Up	EZH2	Promote the growth of HNSCC cells.	CDDP/cetuximab	[116]
HNSCC	POP1-1	HN4, HN30	Up	MCM5	Facilitate the repair of DNA damage	CDDP	[117]
LSCC	FOXD2-AS1	Hep2, TU-212	Up	STAT3	Promote STAT3 transcriptional activity	CDDP	[119]
OSCC	CYTOR	CAL-27, SCC4	Up	ceRNA	Activate CYTOR/LPP axis	CDDP	[76]
OSCC	HOXA11-AS	HSC3, HSC4	Up	microRNA-494	Facilitate tumor growth	Dicoumarol	[56]
OSCC	EGFR-AS1	primary cell cultures	Up	/	Resistance to EGFR TKIs	TKIs	[126]
PTC	GLTC	BCPAP, TPC-1, KTC-1	Up	LDHA	Resistance to RAI	RAI	[123]

HNC: Head and neck cancer; NPC: nasopharyngeal carcinoma; CDDP: cisplatin; HNSCC: head and neck squamous cell carcinoma; LSCC: laryngeal squamous cell carcinoma; OSCC: oral squamous cell carcinoma; EGFR: epidermal growth factor receptor; TKIs: tyrosine kinase inhibitors; PTC: papillary thyroid cancer; GLTC: glycolysis-associated regulator of LDHA post-transcriptional modification; LDHA: lactate dehydrogenase A; RAI: radioiodine.

Substantial evidence supports the dependence of cellular homeostasis on lncRNAs^[125,127]^. However, despite a small fraction of the thousands of expressed lncRNAs potentially having functional roles in cancer cells, the extent of their involvement remains inadequately studied^[128]^. Further research is warranted to explore the role of lncRNAs in various aspects concerning tumor chemotherapy, targeted therapy, and immunotherapy, as well as their impact on the tumor microenvironment^[129,130]^.

## circRNAs IN HNC CELL DRUG RESISTANCE

The discovery of single-stranded covalently closed circular RNA dates back to 1976, with subsequent findings revealing their common presence in both viruses and mammals^[131]^. circRNAs exhibit a diverse range of functions, encompassing their role as protein scaffolds or miRNA sponges, and their capacity for translation into polypeptides^[132]^. The unique structure of circRNAs grants them a longer half-life compared to linear RNA and provides resistance against RNase R degradation^[133]^. Consequently, circRNAs have garnered significant attention as reliable diagnostic and prognostic biomarkers in cancer diagnosis, treatment, and prevention^[13,16,134-137]^.

### circRNAs mediate drug resistance of NPC

Patients with distant metastases of NPC exhibit specific overexpression of circIPO7, and its knockdown enhances sensitivity to CDDP treatment by inhibiting YBX1 nuclear localization^[138]^. Furthermore, studies have reported an association between aging and tumor metastasis and chemoresistance. Notably, circWDR37 facilitates the activation of PKR, leading to the initiation of SASP transcription through NF-κB, thereby promoting metastasis and chemoresistance in NPC^[139]^. A separate study demonstrated the role of circCRIM1 as a ceRNA in promoting metastasis and conferring docetaxel chemoresistance in NPC through the upregulation of FOXQ1. Additionally, the study successfully developed a prognostic model based on circCRIM1 expression and TMN staging to assess the risk of NPC metastasis^[80]^.

### circRNAs mediate drug resistance of HNC

**Figure 3:**
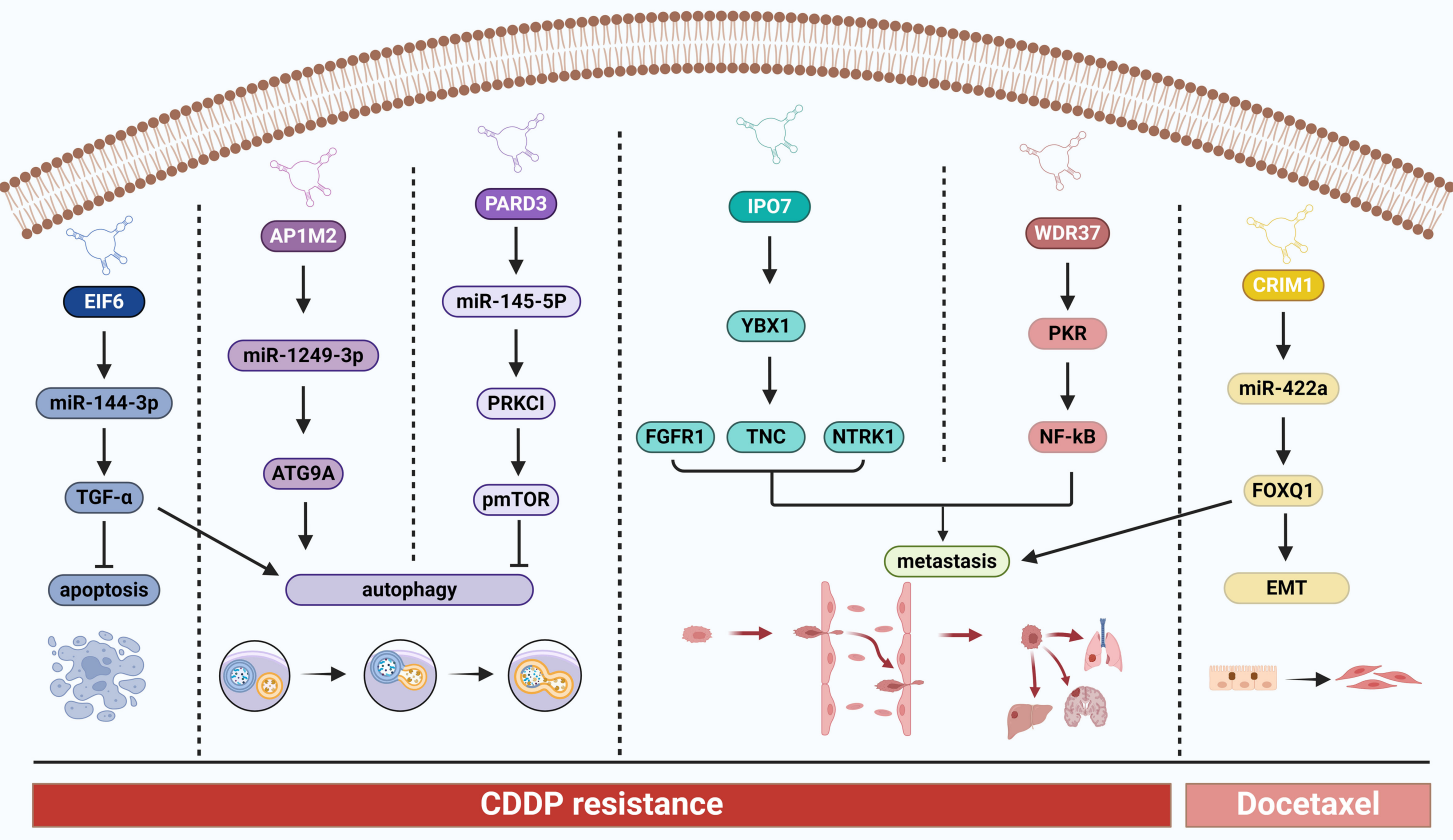
Overview of the main molecular mechanisms of circRNAs in HNC drug resistance. HNC: Head and neck cancer.

CDDP resistance in HNC is strongly linked to autophagy, a process essential for maintaining protein homeostasis, organelle integrity, cellular homeostasis, cell viability, and the degradation/recirculation of various cellular components to the lysosome^[140,141]^. In OSCC, significant upregulation of circAP1M2 leads to the induction of autophagy-associated CDDP resistance via the miR-1249-3p/ATG9A axis^[51]^. In LSCC, circPARD3 hinders autophagy by serving as a sponge for miR-145-5p, which activates the PRKCI/Akt/mTOR pathway, thereby promoting tumor progression and contributing to CDDP resistance^[142]^. TC is characterized by the promotion of autophagy and increased CDDP resistance due to the regulatory effects of circEIF6 on the miR-144-3p/TGF-α axis^[143]^ [Table 4 and Figure 3]. Interestingly, autophagy exhibits dynamic tumor-suppressive or tumor-promoting roles in diverse contexts and stages of cancer development^[144]^. Therefore, further research is needed to better define the specific roles of autophagy in various types and stages of cancer, and to gain a deeper understanding of how tumors rely on autophagy. Furthermore, several studies have demonstrated the reciprocal regulation between circRNAs and N6-methyladenosine (m6A) modifications in HNC, highlighting their potential impact on cancer progression and treatment response^[145-148]^. Additionally, there have been reports indicating the upregulation of circCUX1 expression in radiotherapy-resistant hypopharyngeal SCC patients, and the knockdown of circCUX1 has been shown to enhance the sensitivity of hypopharyngeal cancer cells to radiotherapy^[149]^.

**Table 4:** HNC cell drug resistance-related circRNAs

**Tumor type**	**circRNA**	**Cell line**	**Expression level**	**Target**	**Functions**	**Corresponding drugs**	**Ref.**
NPC	IPO7	CNE2, HNE1, HONE1, SUNE1, HK1, C666-1	Up	YBX1	Promote cell migration, invasion, and cisplatin resistance	CDDP	[138]
NPC	WDR37	S18, S26	Up	PKR	CCND1	CDDP, gemcitabine	[139]
NPC	CRIM1	S18, S26	Up	miR-422a	Promote metastasis and EMT	Docetaxel	[80]
OSCC	AP1M2	CAL27/CDDP	Up	miR-1249-3p	modulate miR-1249-3p-ATG9A axis	CDDP	[51]
LSCC	PARD3	Tu 177, HOK, FD-LSC-1	Up	miR-145-5p w	Activate the Akt-mTOR axis	CDDP	[142]
ATC	EIF6	TPC1, BHT101	Up	miR-144-3p	Regulate miR144-3p/TGF-α axis	CDDP	[143]

HNC: Head and neck cancer; NPC: nasopharyngeal carcinoma; CDDP: cisplatin; CCND1: stimulate cyclin D1; EMT: epithelial-mesenchymal transition; OSCC: oral squamous cell carcinoma; LSCC: laryngeal squamous cell carcinoma; ATC: anaplastic thyroid carcinoma.

## FUTURE PERSPECTIVES

Based on our persistent dedication to this field and thorough literature research, we firmly assert that the following areas of research exhibit dynamism and merit sustained attention.

### Ferroptosis

Ferroptosis, also known as iron-induced apoptosis, is a type of intracellular cell death that relies on iron and is distinguished by the excessive accumulation of reactive oxygen species (ROS) and lipid peroxidation. It is distinct from apoptosis, necrosis, and autophagy^[150,151]^. Numerous studies have reported that tumor cells can enhance their defense mechanisms against oxidative stress by inhibiting ferroptosis, ultimately promoting their survival and drug resistance^[152,153]^. Studies have demonstrated that in NPC, infection with EBV leads to the upregulation of GPX4 expression, resulting in the inhibition of ferroptosis. Consequently, the elevated levels of GPX4 contribute to the progression of NPC and its resistance to chemotherapy through the activation of the TAK1-JNK and IKK/NF-κB signaling pathways. Moreover, clearance of the EBV genome has been shown to enhance the sensitivity of NPC cells to ferroptosis^[154]^. Artesunate exhibits selective cytotoxicity against HNC cells. In specific cases of CDDP-resistant HNC, inhibiting the Nrf2-ARE pathway enhances the sensitivity of these cells to artesunate and reverses their resistance to ferroptosis^[155,156]^. Additional studies have further demonstrated that inhibiting GLRX5 renders CDDP-resistant HNC cells more susceptible to ferroptosis^[157]^. Furthermore, the upregulation of HMGA1 in ESCC serves as a crucial factor responsible for CDDP resistance by suppressing ferroptosis. This effect is achieved through HMGA1’s role in maintaining intracellular redox homeostasis via its assistance to ATF4 in activating SLC7A11 transcription. Inhibition of HMGA1 has been shown to enhance the sensitivity of ESCC to ferroptosis^[158]^. Multiple studies have now established that regulating ferroptosis can impact the effectiveness of cancer treatment and potentially overcome resistance to chemotherapy, targeted therapy, and immunotherapy^[159-164]^. Several non-coding RNAs, including miR-324-3p, miR-375, miR-144-3p, miR-27a-3p, miR-3173-5p, circRNA-101093, and lncRNA-PMAN, have been implicated in the regulation of tumor ferroptosis^[165-168]^. Currently, there are no relevant studies investigating the role of non-coding RNAs in tumor drug resistance through the regulation of ferroptosis specifically in HNC.

### Nanodrugs

CDDP, carboplatin, and oxaliplatin are commonly employed in tumor therapy. Nevertheless, their clinical utility is severely restricted due to the side effects associated with platinum drugs, including poor selectivity, high systemic toxicity, and drug resistance^[169]^. A novel CDDP nanocarrier system with dual targeting properties has been developed. This system selectively attaches to the A54 receptor, which is highly expressed on the cell surface of hepatocellular carcinoma (HCC) cells. Additionally, it utilizes the drug-resistance gene NOR1 shRNA as a piggyback mechanism to enable precise tumor-targeted therapy and overcome drug resistance^[170]^. Numerous studies have also highlighted the effectiveness of platinum nanocarriers in reducing systemic toxicity and overcoming drug resistance^[171-174]^. Currently, there is considerable research focus on strategies involving nanomedicines for regulating ferroptosis, as well as nanomedicines utilizing non-coding RNAs as carriers to target tumors^[85,175-179]^.

### Exosomes

Exosomes are nanovesicles derived from cells, ranging in size from 30 to 150 nm. They are released when multivesicular bodies fuse with the cell surface and play a crucial role in intercellular communication by transporting nucleic acids, proteins, and lipids. Furthermore, exosomes can activate signaling pathways in target cells^[180,181]^. In recent years, there has been significant interest in the role of exosomes carrying non-coding RNA in tumor drug resistance^[93,163,182-185]^. Several studies have reported that intervention with RNA carried by exosomes can potentially overcome tumor chemoresistance^[61,186,187]^. For instance, CAFs emerge as crucial regulators of CDDP resistance in HNC. They achieve this by transporting functional miR-196a from CAFs to tumor cells through exosomes^[62]^. Although our understanding of the role of RNAs carried within exosomes in the mechanisms of drug resistance in HNC is still in the early stages, exosomal circRNAs have emerged as innovative genetic information carriers. These molecules enable communication between tumor cells and cells in the microenvironment, thereby regulating critical aspects of cancer progression. As a result, they contribute significantly to chemotherapeutic drug resistance across different types of cancer. Based on this, synthetic exosomal circRNA holds the potential to introduce new avenues for cancer therapy^[188,189]^.

### Proteolysis-targeting chimeras

Proteolysis-targeting chimeras (PROTACs) are emerging as promising therapeutic modalities for the degradation of disease-causing proteins. They are composed of a ligand that binds to a protein of interest (POI) and another ligand that recruits the E3 ubiquitin ligase. This recruitment induces chemical proximity between the POI and the E3 ligase, resulting in ubiquitination and subsequent degradation of the POI through the ubiquitin-proteasome system^[190-193]^.PROTACs have been developed to target several proteins, including PD-L1, BTK, STAT3, EGFR, MEK1/2, VEGFR2, FLT-3, and SHP2^[194]^. These compounds offer advantages such as high enzymatic reaction efficiency, overall degradation of target proteins, and the ability to target small-molecule non-druggable proteins. Several studies have reported the successful inhibition of HNSCC growth and metastasis using PROTAC technology^[195,196]^. This finding highlights the importance of continued attention to PROTAC and related technologies due to their ability to specifically degrade oncogenic proteins or RNAs^[194,197,198]^.

## CONCLUSION

In summary, chemotherapy, targeted therapy, and immunotherapy are crucial components of integrated tumor therapy. However, drug resistance significantly limits the effectiveness of clinical drug therapy for HNC. Non-coding RNA dysregulation plays a critical role in the known mechanisms of drug resistance in HNC. It is essential to continually focus on new technologies and theories to address the challenges in HNC drug resistance research. This includes the construction or utilization of clinical databases similar to UK biobank^[199]^, and integrating clinical information with multi-omics sequencing data. These approaches aim to establish a robust foundation for further research on integrative oncology treatment^[200]^. Additionally, leveraging ChatGPT automated development and analysis (ADA) technology to build machine learning models based on real clinical trial data^[201]^ can facilitate efficient research on cancer precision treatment, prognosis, and drug resistance markers.

## DECLARATIONS

### Copyright

© The Author(s) 2024.
